# Osteoid osteoma of the distal phalanx of the finger: Case report and review of literature

**DOI:** 10.1016/j.radcr.2024.11.034

**Published:** 2024-12-09

**Authors:** Ashima Kundu, Liana Ysabel Almendras Bautista, Haley Clark, Usman Beg, Amirmasoud Negarestani, Emad Allam

**Affiliations:** Loyola University Medical Center and Loyola University Chicago, 2160 S First Ave, Maywood, IL 60153, USA

**Keywords:** Osteoid osteoma, Distal phalanx, Finger, Benign bone lesions

## Abstract

Osteoid osteoma (OO), a benign bone-forming tumor estimated to account for 3% of all primary bone tumors, rarely occurs in the finger. This case report presents an unusual instance of osteoid osteoma in the finger of a 15-year-old male patient. The lesion was discovered following an initial patient visit for left middle finger pain and swelling for one year without any identifiable injuries.

## Background

Osteoid osteoma (OO) is a benign bone-forming tumor that typically affects young individuals under 30 years of age with a male predominance. It accounts for approximately 10%-14% of all benign bone tumors and 3% of primary bone tumors [[1], [2]]. The characteristic feature of OO is the presence of a nidus, usually a single, round lytic lesion up to 2 cm in diameter, surrounded by an area of reactive sclerosis. These tumors commonly occur in the cortex of long bones, particularly in the diaphyses or metaphyses [3]. Within the hand, OO has been noted to occur in the phalanges in 60% of cases, the carpal bones in 30% of cases. and the metacarpal bones in 10% of cases [4]. The classic clinical presentation includes distinctive night pain that responds well to nonsteroidal anti-inflammatory drugs (NSAIDs). Patients with OO in the hand are found to have long histories of pain and may even present with hypertrophy of the nail bed [5]. Diagnosis is typically suggested through imaging studies, with plain radiography and computed tomography usually sufficient for identification. However, diagnosing OO in the hand is often complicated by the fact that imaging tends to be less sensitive and specific compared to other body parts [6]. OO found in the hand can mimic various other conditions, potentially leading to misdiagnosis or delayed diagnosis and treatment [2,7]. While osteoid osteomas can regress spontaneously over 2-6 years, treatment options include NSAIDs and surgical intervention, with minimally invasive techniques such as radiofrequency ablation and cryoablation now considered the gold standard for surgical treatment. Treatment of OO in the hand may pose challenges due to the relatively high number of vital structures in the hand and smaller sized bones.

## Case presentation

**Fig. 1:**
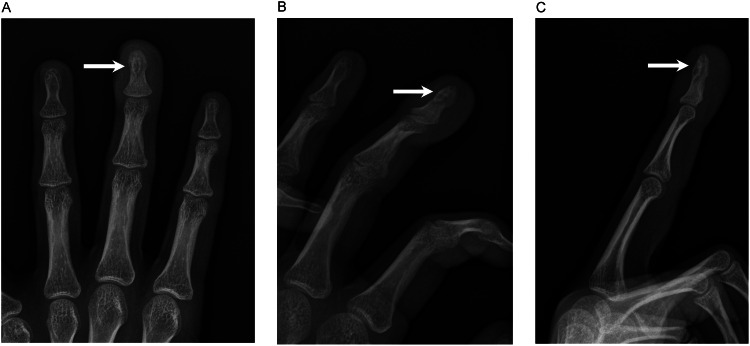
(A, B, C) Frontal, oblique, and lateral radiographs of the left middle finger show a lucent lesion with sclerotic margins in the left middle finger distal phalanx tuft.

**Fig. 2:**
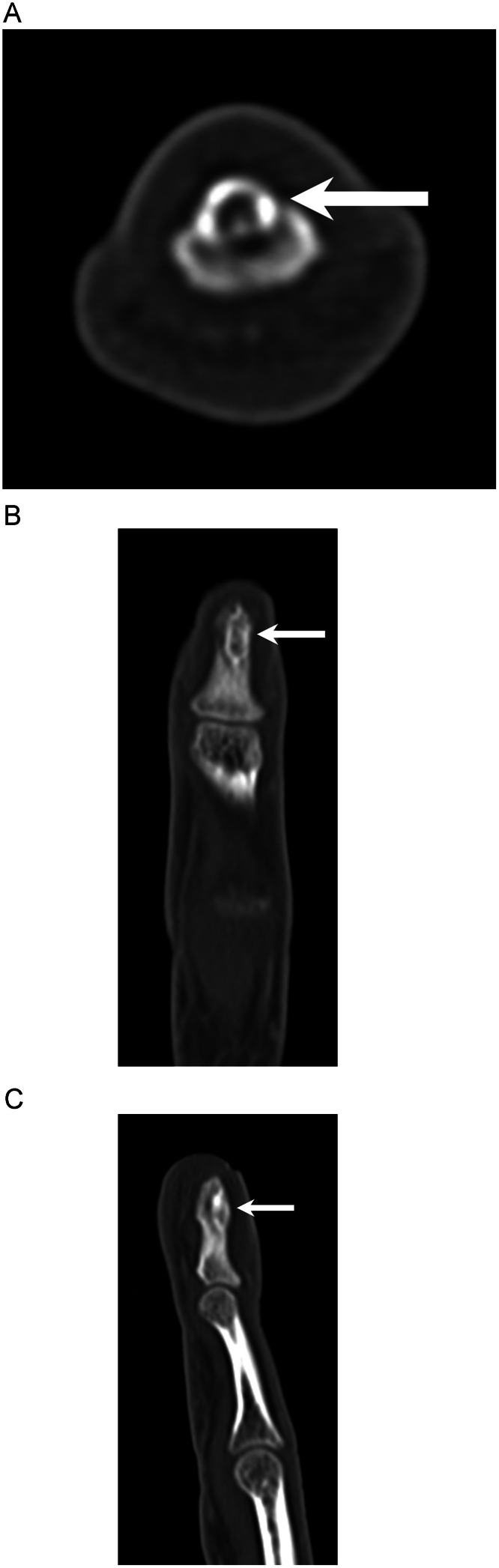
(A, B, C) Axial, coronal, and sagittal CT images of the left middle finger show an ovoid lucent lesion with sclerotic margins in the left middle finger distal phalanx tuft. The lesion measures approximately 6 mm long by 2 mm deep by 3 mm transverse. The lesion appears expansile and is centered in the dorsal cortex with dorsal bony protuberance.

**Fig. 3:**
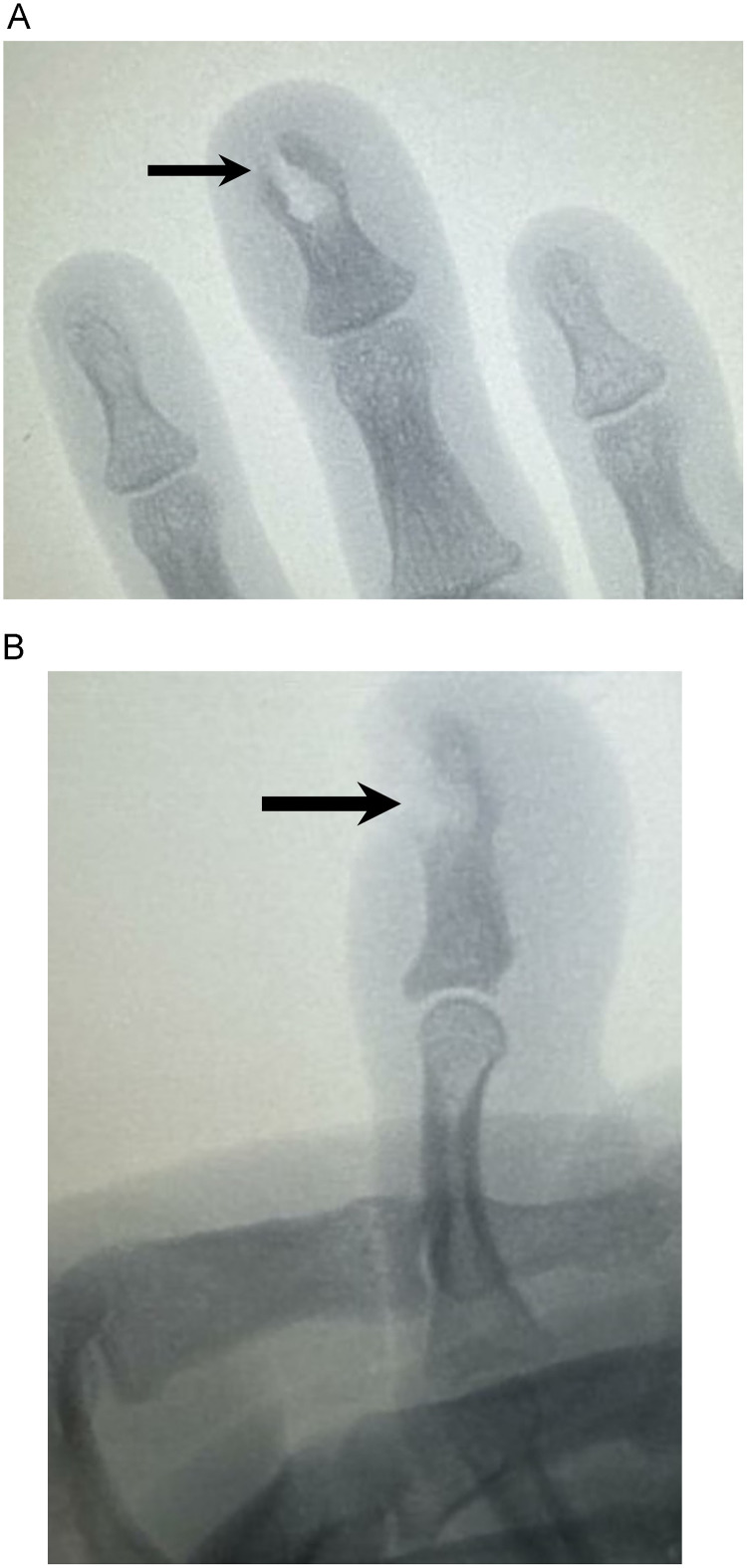
(A, B) Frontal and lateral intra-operative fluoroscopic images show a defect in the left middle finger distal phalanx tuft at the site of surgical excision.

A 15-year-old male patient in otherwise good health presented with a one-year history of persistent pain and swelling in his left middle finger. Physical examination revealed localized swelling and tenderness of the distal phalanx, with full range of motion in the interphalangeal joints. The patient described pain in the distal phalanx with nocturnal exacerbations which was relieved by NSAIDs. He did not have any history of trauma or infection. Radiographs and CT revealed a subcentimeter ovoid expansile lucent lesion with sclerotic margins in the middle finger distal phalanx tuft centered in the dorsal cortex, subungual in location (Fig. 1, Fig. 2). Differential diagnosis based on imaging included subperiosteal hematoma, osteomyelitis, enchondroma, and osteoid osteoma. Lack of a soft tissue lesion made glomus tumor unlikely. The patient subsequently underwent surgical excision of the lesion. The nail was removed and the nailbed was split longitudinally for this surgery (Fig. 3). Final pathology was consistent with an osteoid osteoma. The patient reported complete resolution of finger pain after the surgery.

## Discussion

This case report presents a unique instance of OO in the finger of a 15-year-old male patient, highlighting the challenges associated with diagnosing OO as it appears in an atypical location. The presentation in this case aligns with the typical age range and gender predilection of OO, which predominantly affects males under 30 years of age. However, the location in the finger is rare, which may lead to delayed or missed diagnosis.

The patient's clinical presentation of persistent pain with nocturnal exacerbation and responsiveness to NSAIDs aligned with the classical signs of OO and provided clues towards a diagnosis of OO. Pain associated with OO is thought to be associated with prostaglandin E2 (PGE2) production by OO which has been found to be up to a thousand times greater than normal levels [8]. Despite the patient's clinical profile fitting that of OO, symptoms such as pain and nail deformities may easily be misinterpreted as infection or post-traumatic conditions [9]. Such symptoms in atypical locations may lead to difficulty in accurate and timely diagnosis of the lesions. Furthermore, patients have been reported to attribute their symptoms to trauma [10], creating potential for OO to not be recognized. The 1-year duration of symptoms before diagnosis in this case underscores the potential for delayed recognition of OO in the hand, a common issue noted in the literature.

Imaging played a crucial role in the diagnosis of this case. While plain radiography can help detect lesions, advanced imaging such as CT and MRI can further define the lesion and help guide surgical planning. The final diagnosis of OO was confirmed post-procedure by the pathology in this case.

The decision to proceed with surgical excision rather than minimally invasive techniques like radiofrequency ablation was determined by the location and size of the lesion. While radiofrequency ablation has become the gold standard for OO treatment in many cases, the complex anatomy of the hand and the proximity to skin and neurovascular structures can make this approach challenging in distal phalanx lesions [11]. Successful radiofrequency ablation for treatment of osteoid osteoma of the proximal phalanx of the middle finger has been previously reported [12]. The surgical approach, including nail removal and longitudinal splitting of the nailbed, demonstrates the meticulous technique required for adequate exposure and complete excision of the tumor in the distal phalanx in this case.

The resolution of pain following surgery confirms the effectiveness of surgical excision for OO in this challenging anatomical site. This outcome is consistent with reported success rates for surgical treatment of OO, which range from 88% to 100% [7]. This case highlights the importance of considering OO in the differential diagnosis of persistent digital pain, even in atypical locations such as the distal phalanx. It also demonstrates that while minimally invasive techniques have gained popularity, surgical excision remains a viable and effective option for OO treatment, particularly in anatomically challenging locations where complete removal of the nidus is crucial for symptom resolution and prevention of recurrence.

## Conclusions

This case report reviews the successful diagnosis and surgical management of an osteoid osteoma in an atypical location - the distal phalanx of the middle finger of a 15-year-old male patient. The case underscores the importance of maintaining a high index of suspicion for osteoid osteoma in cases of persistent digital pain and the value of imaging in aiding diagnosis. Due to the diagnostic challenges posed by the tumor's location, a combination of clinical presentation, imaging studies, and histopathological examination were all necessary to accurately come to the diagnosis of OO of the distal phalanx. Successful surgical excision resulted in symptom resolution, proving to be the treatment of choice in this case due to the small size and anatomically challenging location of the OO which precluded the typical radiofrequency ablation treatment option. This case contributes to the limited literature on osteoid osteomas in the distal phalanx and emphasizes the need for a multidisciplinary approach in managing such cases. Future research should focus on optimizing diagnostic strategies and exploring minimally invasive treatment options for osteoid osteomas in small bones.

## Patient consent

Informed consent for this case was obtained from the patient's parent.
